# A multimodal dataset for human robot collaborative systems: Experimental data

**DOI:** 10.1016/j.dib.2025.112234

**Published:** 2025-11-04

**Authors:** Shakra Mehak, Aayush Jain, John D. Kelleher, Michael Guilfoyle, Philip Long, Maria Chiara Leva

**Affiliations:** a Pilz Ireland, Cork Campus, Ireland; b Technological University Dublin, Dublin D07 EWV4, Ireland; c Trinity College Dublin, Ireland; d Atlantic Technological University, Galway, Ireland

**Keywords:** Multimodal, Human-Robot Interaction (HRI), Collaborative Applications, Human Robot Interaction, Robotics

## Abstract

Human-Robot Interaction (HRI) systems are becoming increasingly integral to collaborative industrial and service environments. However, understanding human performance within such settings, particularly in Programming by Demonstration (PbD) frameworks, remains a challenge due to limited availability of comprehensive datasets. This study presents a multimodal dataset designed to assess human performance in HRI applications. The dataset includes both objective and subjective measures collected using various tools within a PbD framework. The data is collected to assess the overall system performance, with a focus on the effectiveness of PbD methods in improving robot learning process. The performance attributes encompass programming efficiency, cognitive workload, usability, and ergonomic assessment of human posture in physical HRI settings. Using tools such as a robot manipulator (UR10e), a vision -based motion tracking system, eye- tracking glasses, NASA Task Load Index (NASA-TLX) and system usability scale (SUS) questionnaire, we recorded interactions between human and robot comprising-robot trajectories, human motion trajectories, participant eye tracking metrices, and participant subjective responses. The data was collected in an experimental setting involving two kinaesthetic robot teaching tasks, under four feedback conditions that provide performance related insights and actionable instructions to improve participant performance. A total of N=28 participants performed three trials of each task using developed Human-Machine Interface (HMI). This dataset is valuable for advancing human-robot teaching interaction and is applicable for developing adaptive HRI systems. The dataset can serve as a significant resource for researchers and machine learning engineers aiming to develop advance HRI systems, improve PbD algorithms, train HRI based machine learning models and facilitate further studies in safety and ergonomic assessment applications in robot learning and collaboration.

Specifications Table

**Table:** 

Subject	*Human Machine Interaction*
Specific subject area	*Human Robot Interaction, Human Robot Collaboration, Human Factors, Programming by demonstration, Machine Learning*
Type of data	*Raw, Filtered, Numerical* *CSV Files (.csv), Excel(.xlsx), Table* *Robot Trajectories, Cognitive data, Human Posture data via MediaPipe and Questionnaires responses, Participant Demographic information*
Data collection	The dataset contains task performance, behavioural, and cognitive data collected from N=28 participants performing kinaesthetic-based programming tasks under four feedback conditions and three feedback modalities: no-feedback, visual feedback, descriptive feedback and mixed feedback. Each condition simulated different HRI settings within PbD tasks. Feedback for the programming task was provided via an HMI, displaying real-time information based on the feedback condition assigned to each task. Participants performed two tasks - object pick-and-place and object sliding, each lasting 10-13 minutes. The participant data is anonymized, and the consent was obtained on the test day. All the participants received training by the researchers and were given 7 minutes practice session to get familiar with designed system. They were asked to wear a portable Tobii pro eye tracker during the course of experiment. After completing the tasks, participants were required to fill out the NASA-TLX and SUS questionnaires. The data collected is initially stored in ROSBags and t*sv* formats which are later extracted to csvs. and xlx. formats*.*
Data source location	*Irish Manufacturing Research, Mullingar, Ireland*
Data accessibility	***Repository Name*** *Zenodo* Data identification number: 10.5281/zenodo.15466479 Direct URL to data: Multimodal-Human-Robot-Teaching-Dataset Instructions for accessing these data: The dataset is available on Zenodo at 10.5281/zenodo.15466479. Download the compressed file and extract it to access the structured folders containing eye-tracking, motion, and robot trajectory data.
Related research article	[1] Mehak, S., D Kelleher, J., Long, P., Guilfoyle, M., & Leva, M. C. (2023). Understanding and Quantifying Human Factors in Programming from Demonstration: A User Study Proposal. [2] Jain, A., Long, P., Villani, V., Kelleher, J. D., & Leva, M. C. (2024, May). CoBT: Collaborative Programming of Behaviour Trees from One Demonstration for Robot Manipulation. In 2024 IEEE International Conference on Robotics and Automation (ICRA) (pp. 12993-12999). IEEE.

## 1 Value of the Data

• Data needed for assessing HRI in teaching and PbD is scarce. This dataset contributes to the growing body of research, providing valuable insights into HRI studies and robot learning where operators train robots or act as supervisors.
• The dataset combines robotic trajectories, human postures data in kinesthetic settings and cognitive metrics to offer a broader view of cognitive, physical and operational factors in robot programming environments. This multimodal approach can help the researcher to design user-centric programming interfaces that consider both the human and robot perspectives.
• The dataset can serve as a reliable baseline for testing and validating machine learning models in fields such as anomaly detection in robot programming and task performance optimization. Moreover, it can also be used to design adaptive HRI systems that can adjust their behavior based on user performance and cognitive load.
• Researchers can use this data to train models that dynamically adapt feedback and interaction methods for intelligent and responsive robotic systems. Specifically, demonstration data can be utilized to monitor performance and identify suboptimal paths. Human posture data can reveal teaching strategies and potential risks in human-robot interaction (HRI) that may pose ergonomic concerns. This data can enable adaptive robots to modify their movements or adjust the height of their interactions upon detecting signs of strain. Additionally, eye-tracking data offers insights into an individual's focus of attention and can activate adaptive visual prompts or highlight objects when confusion is detected. The integration of these three types of data facilitates the development of context-aware machine learning models capable of adjusting robot behaviour and interface feedback in real-time. This method can contribute to research on user-aware adaptation in HRI.
• HMI designers can leverage this data to understand feedback impact or feedback modalities to improve the efficiency of interfaces in human-machine interaction studies.
• Beyond robotics, the dataset is valuable for human factor studies to quantify human behaviors, and to develop human performance models

## 2 Background

PbD enables humans to teach robots new tasks by using various demonstration modalities in HRI settings [3]. One of such modality is kinaesthetic teaching, which allows the operator to physically guide the robot through desired motions. This intuitive approach lowers the barrier to robot programming, empowering non-expert users to effectively instruct the robot. A critical factor in enhancing the PbD process is the feedback provided to the human operators during the teaching. Different feedback modalities, performance-related metrics, and actionable instructions can influence the quality and effectiveness of teaching. However, optimizing such interactions requires a comprehensive dataset that not only captures robot policy but also provides a deeper understanding of the human factors involved.

Existing datasets often overlook comprehensive performance-related data that illustrate how humans interact with robots in supervisory or teaching roles within PbD framework. To address this gap, we conducted an experimental study with N=28 participants, resulting in a rich multimodal dataset. The dataset includes human cognitive data, human and robot joint kinematics, and subjective participant feedback. This dataset aims to deepen our understanding of how various feedback modalities affect human teaching behavior and system performance, ultimately supporting the development of adaptive HRI and intuitive robot programming methods.

## 3 Data Description

*This section provides an overview of the dataset, detailing the structure, types and usability of data collected.*

### 3.1 *General Overview*

The data repository is organized using a data-centric hierarchical structure, as summarized in Table 1 The dataset is divided into four primary data types based on the modality of data collected during human-robot teaching experiments:

**Table 1:** Overview of all data streams recorded in the multimodal dataset.

Data Category	Variables Group	Variables	Description
*RobotTrajectory Dataset*	Timestamp	*Time*	Timestamp of each data frame.
	End-Effector Pose and Orientation	*Cartesian coordinates (X, Y, Z); Quaternion values (Qw, Qx, Qy, Qz)*	6-DOF pose data of the robot end-effector representing position and orientation of the tool center point during demonstrations.
	Gripper State	*Gripper*	Single scalar value representing degree of grip (0 = open, 1 = fully closed).
	Robot Joint Angles and Velocities	*robot_elbow_joint, robot_shoulder_lift_joint, robot_shoulder_pan_joint, robot_wrist_1_joint, robot_wrist_2_joint, robot_wrist_3_joint, Vrobot_elbow_joint, Vrobot_shoulder_lift_joint, Vrobot_shoulder_pan_joint, Vrobot_wrist_1_joint, Vrobot_wrist_2_joint, Vrobot_wrist_3_joint*	Joint positions and joint velocity values. Recorded joint angles representing full 6-DOF configuration
	Object Pose	*Object_ID, Object_X, Object_Y, Object_Z, Object_Qw, Object_Qx, Object_Qy, Object_Qz*	Spatial pose and orientation of the manipulated object.
*EyeTracking* *Dataset*	Participant Information	*Participant_Condition_Task, TOI, Interval*	Participant ID and experimental condition details, trial, and interval of recording
	AOI Details	*Media, AOI,* *Number_of_saccades_in_AOI, Start_AOI, Landing_AOI*	Area of Interest annotations generated in Tobii Pro Lab
	Fixation Metrics	*Number_of_fixations,* *Average_duration_of_whole_* *fixations*	Fixation duration statistics and counts
	Pupil Metrics	*Average_pupil_diameter,*	Average pupil size per interval
	Temporal Measures	*Average_duration,* *Minimum_duration, Maximum_duration,*	Duration measures of fixations across AOIs
	Saccade metrics	*Saccade_direction, Average_velocity,* *Peak_velocity, Saccade_amplitude*	Direction and kinematic features of saccades
	Event details	*Event_type, Validity,* *EventIndex, Start, Stop, Duration*	Type and timing of gaze events (fixation, saccade) and data quality
	Positioning	*Start_position_X/Y, Landing_position_X/Y*	Gaze coordinates at start and end of event
	Visit-level metrics	*Average_duration_of_Visit, Number_of_saccades_in_AOI*	Duration and frequency of gaze visits to AOIs
Human Motion Dataset	3D Landmarks	*Landmark_X_11–24, Landmark_Y_11–24, Landmark_Z_11–24*	3D coordinates of tracked upper-body landmarks from mediapipe (indices 11–24) representing shoulder, elbow, wrist, and hand joint positions captured from the human demonstrator.
*Questionnaire Response*	Usability Metrics (SUS)	*SUS-Q1 to SUS-Q10*	Ten System Usability Scale items scored on a 5-point Likert scale.
	Workload Metrics (NASA-TLX)	*Mental Demand, Physical Demand, Temporal Demand, Performance, Effort, Frustration*	Subjective workload assessment values.

***RobotTrajectory_Dataset*** *: Records the robot's end-effector positions and trajectory data during task execution, extracted from the robot controller.*

**Fig. 1:**
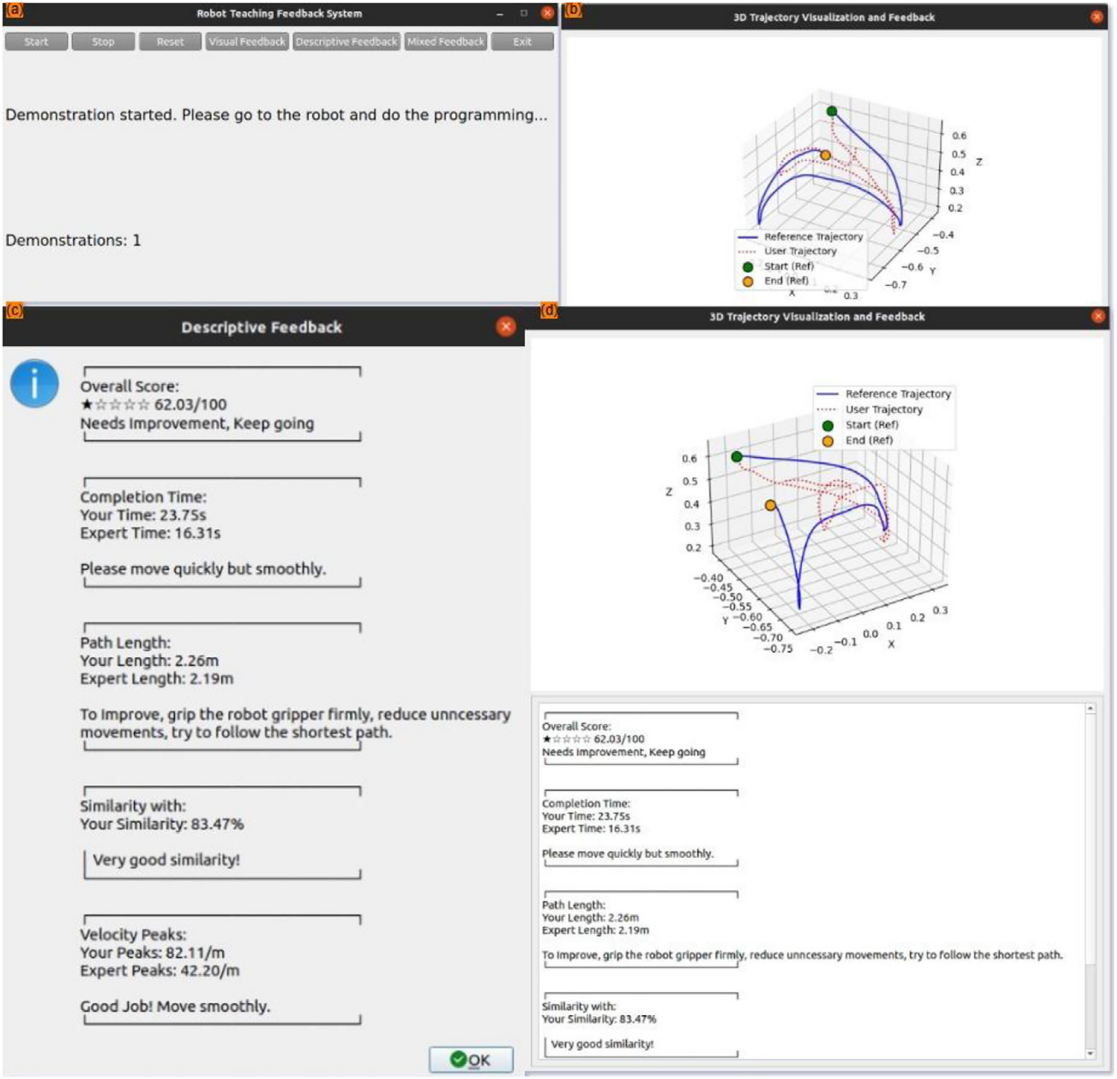
Screenshot of HMI with various feedback types: (a) home screen, (b) visual feedback, (c) descriptive feedback, (d) mixed feedback.
    • Visual Feedback (VF)**:** Participants received real-time graphical feedback through the developed HMI, as shown in Fig. 1b. This included 3D- robot trajectory alignment with optimal trajectory, helping users assess their task performance visually.
    • Descriptive Feedback (DF)**:** Participants received detailed, text-based feedback on various performance metrics such as task completion time, path length, motion smoothness, and similarity index with optimal trajectory. Furthermore, they were provided with actionable suggestions highlighting areas that require improvements.
    • Mixed Feedback (MF)**:** This condition combined both visual and descriptive feedback modalities. Participants received visual cues along with descriptive instructions on the HMI.
  b. **Kinesthetic Teaching Tasks**
    To assess human performance in PbD settings, two kinesthetic programming tasks (as shown in Fig. 2) were designed to reflect typical actions in HRI scenarios. Each participant performed three trials of both tasks.
    • **Task 1 Object Pick and Place:**
      Participants guided the robot to pick an object from a designated location (the green area shown in Fig. 2a) and place it at a target position (the red area shown in Fig. 2a). This task requires precise control to ensure accurate object grasping and placement. The objective of this task is to assess the participant’s ability to demonstrate fundamental manipulation skills and evaluate how different feedback/no feedback effects the precision and efficiency of robot programming process in PbD.
    • **Task 2 Object Sliding:**
      In this task, participants guided the robot to slide an object/the cube over the predefined area on the slider (the red area shown in Fig. 2b) from the initial location (the green area shown in Fig. 2b). Successful execution required consistent force application and directional control to maintain continuous contact between the object and the slider throughout the motion.

**Fig. 2:**
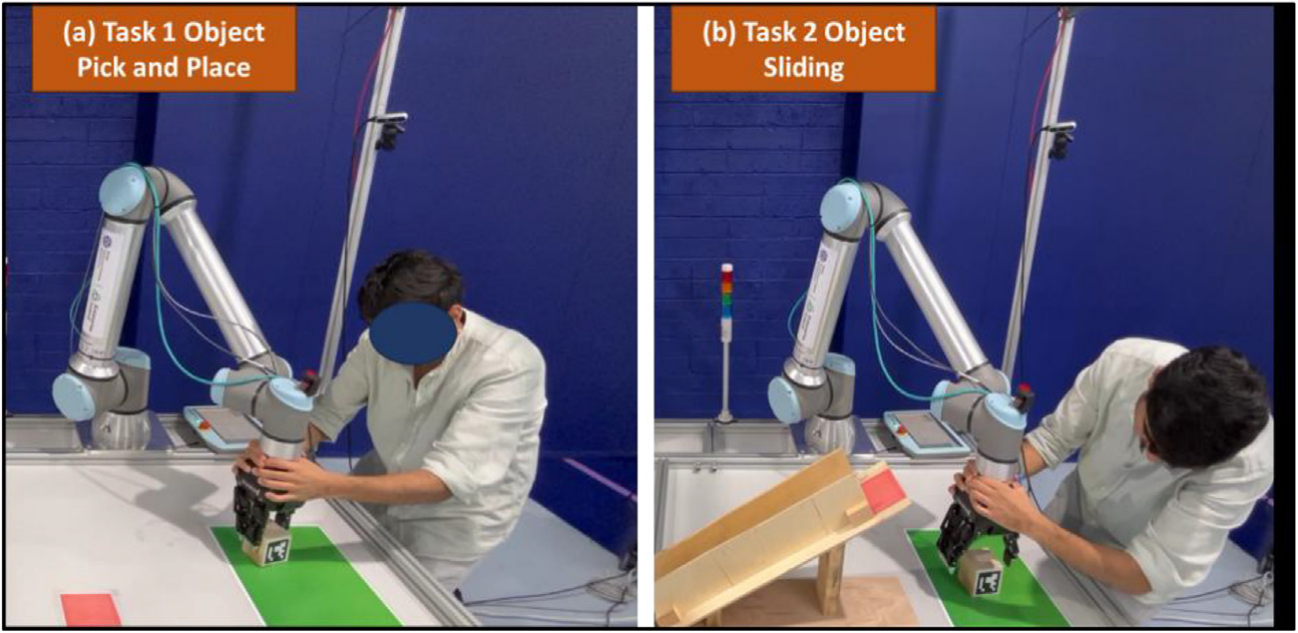
The two kinesthetic programming tasks: (a) object pick and place and (b) object sliding.
  c. **Participant IDs**
    Each participant was allocated a unique identifier (e.g., Participant_01 to Participant_28) to maintain anonymity and confidentiality throughout the study. The identifiers were assigned systematically without linking it to any individually identifiable information.
  d. **Demographic Data:** As a part of pre-experiment questionnaire, we first collected participant’s demographic data to contextualize our experimental data. This included details such as age, gender, educational background and prior experience with robots. Participants also self-reported their familiarity and prior experience with robots to understand their level of expertise. This information allows analysis of how the educational and demographic variables impact on participant’s ability to program the robot and adapt to HMI.
  e. **Robot Demonstration Data**
    The robot demonstration data was collected using the onboard sensors of a UR10e robotic arm. All the recordings initially stored in ROS (Robotic operating system) bag files and later extracted into CSV files for analysis. The demonstrated trajectories were captured by tracking the robot’s end-effector pose, comprising: cartesian coordinates (X, Y, Z), the quaternion values (Qw, Qx,Qy, Qz). The gripper finger position was recorded as a single data point representing the degree of grip. In addition, robot joint positions were recorded including the robot_elbow_joint, robot_shoulder_lift_joint, robot_shoulder_pan_joint, robot_wrist_joint, robot_wrist_2_joint and robot wrist_3_joint. Corresponding joint velocities were also recorded to understand the dynamics of the robot’s movements. Finally, the object tracking data was recorded which consists of both the object identifier via maker IDs and their respective poses. All the data is time-stamped providing information of task execution accuracy over time.
  f. **Cognitive Data (Eye-Tracking Data)**
    To collect human cognitive data points, we used head mounted eye-tracking glasses (Tobii Pro Glasses 3) that can measure the participants pupil size and eye movements. The raw eye-tracking data files were processed in Tobii Pro Lab software, and the interested metrics were extracted for the analysis. The metrics included participant’s gaze point, fixation duration, blink rate and pupil dilation during the task performance including both demonstration phases and feedback interpretation. In addition, AOIs were generated in Tobii Pro Lab software to capture participant attention to key task elements such as the robot, manipulated object, and workspace. AOI mapping was performed semi-automatically within the Tobii software, resulting in temporally annotated gaze data that indicate when and for how long attention was directed to each region. This enables analysis of attention allocation patterns across the four feedback conditions.
    The dataset contains some missing values due to blinks, calibration drift, or temporary signal loss. These characteristics reflect the practical conditions of multimodal HRI experiments and should be considered during preprocessing. The resulting eye-tracking metrics enable detailed examination of how different feedback mechanisms influence operator attention and performance during robot teaching interactions.
  g. **Human Joint Motion Data**
    During task execution, participant motion data were recorded using the MediaPipe framework (Kim et al., 2023), which provides real-time 3D pose estimation. A subset of 14 landmarks focusing on the upper body (e.g., shoulders, elbows, and wrists) was extracted, as these joints are most relevant for analyzing kinesthetic teaching. The raw landmark data were stored in ROS bag files and later exported into CSV format. Each row represents a frame, and each column corresponds to the X, Y, and Z coordinates of a tracked landmark.
    During preprocessing, consistent column labelling was applied to indicate the axis and landmark index. Short gaps caused by occlusions or tracking errors were imputed using linear interpolation to preserve trajectory continuity. Although interpolated samples were not separately annotated, interpolation was applied conservatively and verified through visual inspection to minimize distortion of natural movement dynamics. This refinement improves usability and enables reliable analysis of posture, ergonomics, and movement quality during kinesthetic teaching tasks. Fig. 3 illustrates a participant performing a teaching task with the collaborative robot. The blue markers represent detected upper-body joints obtained from the human posture tracking system, which were used to extract 3D landmark coordinates.

• **EyeTracking_Dataset**: Includes raw gaze coordinates, fixation data, pupil diameter, and Area of Interest (AOI) metrics collected using the Tobii Pro Glasses 3.
• **HumanMotionTracking_Dataset**: Contains body posture and movement data captured using a vision-based motion tracking system.
• **QuestionnaireResponse_Dataset**: Consists of subjective responses, including SUS scores and NASA-TLX responses.
  a. Condition Settings
    The data was collected under four feedback conditions: one No Feedback (NF) and three with performance-based feedback modalities- Visual Feedback (VF), Descriptive Feedback (DF) and Mixed Feedback (MF). Each feedback type was presented to the participant on the HMI screen as illustrated in Fig. 2. The study was designed to evaluate the impact of these feedback on kinesthetic -based teaching performance in PbD framework.
    • No Feedback (NF)**:** This condition served as a baseline to assess participant’s natural performance without any external guidance or cues, as shown in Fig. 1a.

**Fig. 3:**
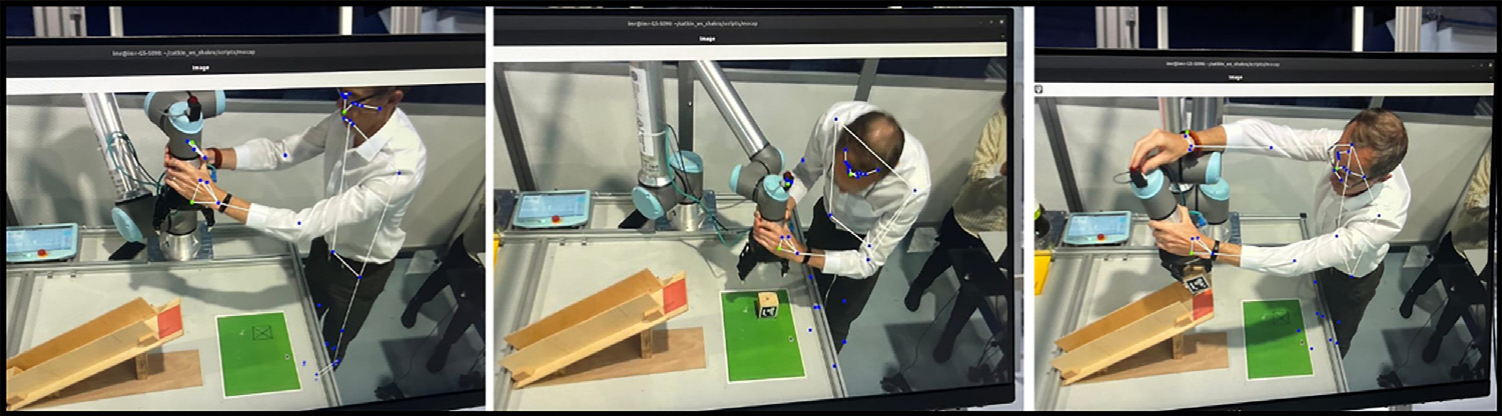
Participant engaged in a kinesthetic teaching task, demonstrating real-time motion cap-ture with MediaPipe’s skeleton overlay visualizing joint and posture data.
  h. **Questionnaire Data**
    • **NASA-TLX:** Following task completion**,** the participants were asked to fill out the questionnaire. It includes multidimensional measures consisting of six key aspects: workload and mental demand, physical demand, temporal demand, performance, effort and frustration level. The responses help to identify factors contributing to participant’s mental and physical workload and allow for a detail analysis of how feedback/no feedback conditions affect participant attention and performance.

**SUS:** The SUS questionnaire is utilized to assess the overall usability of the PbD framework. This state-of-the-art questionnaire consists of ten items that measure various facets of usability, including ease of use, the need for external or additional support and the likelihood of user satisfaction.

### 3.2 Cross-modality data characteristics

The aim of the dataset is to analyse two major aspects: the quality of the demonstration and the physiological data to understand the human performance during PbD. Therefore, the multimodal dataset differs in its temporal alignment across modalities as they are meant to analyse independently. Robot demonstration data are fully time-stamped within ROS, which enables precise analysis of trajectories and joint states over time. Human motion data were also recorded in ROS; however, they do not include timestamps. As a result, these data cannot be aligned frame-by-frame with the robot recordings. They remain suitable for independent analyses such as posture assessment, ergonomic evaluation, and task-level comparisons. Eye-tracking data were collected separately using Tobii Pro Lab and can be associated with the demonstration data through task-event markers, including the type of feedback presented during each condition. Questionnaire responses complement the behavioural and physiological measures by providing subjective assessments of workload and usability.

## 4 Experimental Design, Materials and Methods

### 4.1 Study design

The study was designed as within-participant experiments to assess human performance in robot programming within PbD framework and in HRI settings. The experiments systematically varied feedback conditions which served as the independent variable and performance metrics as dependent variables. To reduce potential learning effects, the feedback conditions were randomized for each participant. Each participant was given three trials to perform both the tasks under four feedback conditions. All participants provided informed consent before the execution.

The experiments were conducted in Irish Manufacturing Research Ireland, as part of Collaborative Intelligence for Safety-Critical Systems (CISC) project. Ethical approval for this study was obtained from the Technological University Dublin Ireland under application REC-20-52B and an internal ethics board within CISC network to ensure that the experimental procedures adhered to the standards of research ethics and participant safety.

### 4.2 Participants

Participants were recruited as volunteers through internal industrial and academic networks and included professionals from diverse backgrounds such as engineering, computer science, and social sciences The inclusion criteria required participants to be over 18 years old with normal or corrected-to-normal, and to have no prior professional experience in robot teaching.The exclusion criteria counted on any history of neurological disorder and any professional robot teaching experience to avoid biasness. A total of 28 participants took part in the experiments, comprising 7 females and 21 males.

### 4.3 *Procedure*

*The experiment is executed in five steps such as preparation, orientation session, task execution, subjective feedback and debriefing.*

• **Preparation:** Participants were screened based on inclusion criteria and the consent form was obtained. The researcher has set up and calibrated the devices to ensure data collection.
• **Orientation Session:** They were provided with a tutorial on the experiment details including experimental setup, a general introduction to PbD framework, task related information and how to use the feedback system and robot. They also had the opportunity to do 10-15 minutes practice sessions to familiarize themselves with the overall setup and tasks.
• **Task Execution:** In this step, they were asked to perform the tasks using the HMI and robot manipulator. During the execution, they were required to wear head mounted eye tracker glasses. Performance and physiological data were collected in this step.
• **Subjective Feedback and Debriefing**: After executing the tasks, participants filled in NASA TLX and SUS questionnaires. The experiment was completed with a debriefing session where they can discuss their experience with the researcher.

### 4.4 Experimental setup and apparatus

The apparatus for the experiments involved different equipment and technologies to facilitate data collection.

**Robotic System:** A UR10e collaborative robotic arm equipped with a gravity compensation controller and RGB-D vision sensor with aruco markers for object detection was used for kinesthetic teaching.

**Human Machine Interface:** The experiment used an intuitive HMI that enables participants to start programming tasks and show feedback in different modalities. The interface supports different feedback conditions and allows participants to see how they performed the programming task to improve their performance in subsequent trial.

**Eye Tracker:** Tobii Pro glasses 3 was used to record detailed eye movement data. It captured data on where participants were looking and their pupil sizes. It was used to record and assess how participant directed their attention while task performance and responding to various feedback modalities. This enabled us to analyze the duration and focus of gaze on specific elements of the interface and interacting with robot.

**Fig. 4:**
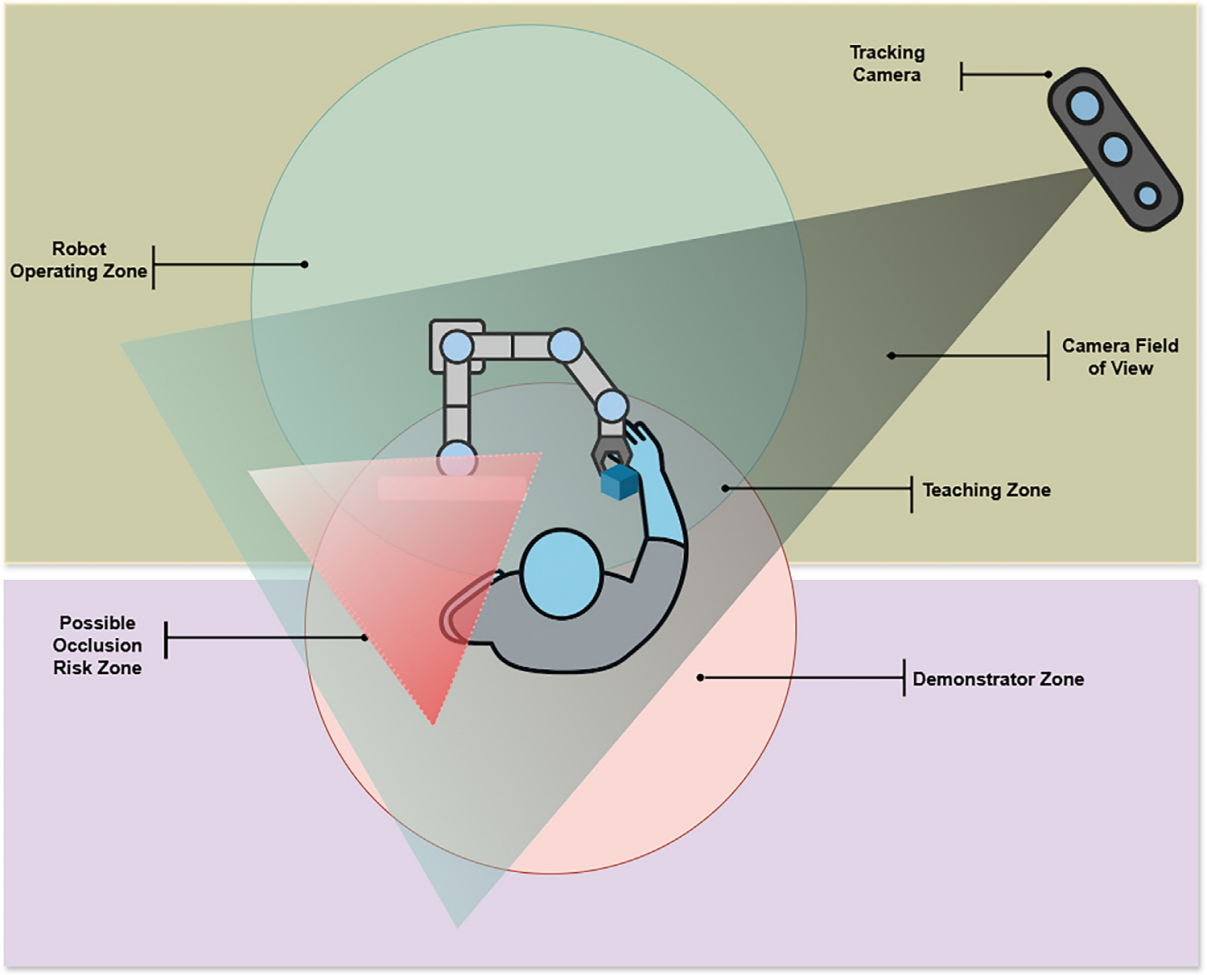
Schematic top-view of the experimental setup showing the robot operating zone, demonstrator zone, teaching zone, and camera field of view with possible occlusion risk areas.

**Motion Capture System:** The experiments employed Google’s MediaPipe technology (Kim et al., 2023) for real-time tracking of participant’s joints movement to capture detailed motion data. Fig. 4 shows the experimental setup of motion capture setup, highlighting the tracking camera’s field of view, robot operating zone, demonstrator zone, and potential occlusion risk areas during teaching interactions.

### 4.5 Software and codes

The source code developed for data extraction and computation is publicly available at [GitHuB].

## Limitations

The dataset presents some limitations inherent to multimodal human–robot interaction experiments. First, the eye-tracking data were not temporally synchronized with the robot and motion-tracking systems, which may reduce the precision of integrated cross-modal analyses. Second, occasional occlusions during human motion tracking resulted in missing landmarks that required interpolation to maintain trajectory continuity. As interpolated samples were not explicitly annotated, users are advised to interpret high-frequency motion metrics (e.g., velocity or smoothness) with caution. Additionally, sporadic sensor dropouts introduced non-uniformly distributed missing values across participants and tasks, reflecting the practical challenges of real-time multimodal data collection.

Despite these constraints, the dataset remains highly valuable for investigating human–robot teaching interactions. It enables the analysis of human teaching behaviors, kinesthetic guidance patterns, and motion characteristics under realistic experimental conditions. The data can also support the development and validation of algorithms for multimodal data fusion, adaptive feedback generation, and robust motion modeling. Users are encouraged to apply appropriate temporal alignment, interpolation, or data exclusion strategies based on their analytical objectives.

## Ethics Statement

Participants received a comprehensive briefing about the experiments and were provided with an information sheet detailing the procedures and requirements of the experiments. They signed a consent form after reviewing experiment information sheet. The ethics application along with the consent form and information sheet received approval from internal Ethics Committee of the Collaborative Intelligence for Safety-Critical Systems and from the ethical review committee of Technological University Dublin, Ireland under the approval number REC-20-52b.

## CRediT Author Statement

**Shakra Mehak:** Conceptualization, Methodology, Software, Data curation, Investigation, Writing- Original draft preparation. **Aayush Jain:** Conceptualization, Software, Reviewing and Editing **John D. Kelleher**: Supervision, Reviewing and Editing, **Michael Guilfoyle:** Supervision, Validation, Reviewing and Editing, Project Administration **Maria Chiara Leva:** Conceptualization, Methodology, Supervision, Reviewing and Editing, Funding Acquisition

## Data Availability

ZenodoMultimodal-Human-Robot-Teaching-Dataset (Original data)
